# Cloacal Malformation with Associated Urethral Atresia

**DOI:** 10.1055/s-0043-1761206

**Published:** 2023-02-07

**Authors:** Tamador Al-Shamaileh, Laura Tiusaba, Shimon Eric Jacobs, Teresa Lynn Russell, Elizaveta Bokova, Hans G. Pohl, Briony K. Varda, Christina Ho, Christina Feng, Andrea Badillo, Marc A. Levitt

**Affiliations:** 1 Department of General Surgery, King Hussein Cancer Center, Amman, Jordan; 2 Division of Colorectal and Pelvic Reconstruction, Department of Surgery, Children's National Hospital, Washington, District of Columbia, United States; 3 Department of Urology, Children's National Hospital, Washington, District of Columbia, United States

**Keywords:** urethra atresia, cloaca, rectal patch

## Abstract

**Introduction** Cloacal malformations comprise a heterogeneous group of anomalies that are considered the most complex anorectal malformations (ARMs) in females. Precise evaluation to identify the unique anatomy prior to reconstruction with collaboration between colorectal surgeons, urologists, and gynecologists is vital. Here, we present a rare anatomical variation in a patient with a cloacal malformation which affected operative and postoperative management.

**Case description** A 6-year-old female with cloaca who underwent colostomy, vaginostomy, and vesicostomy as a newborn presented for reconstruction. Her VACTERL workup was negative except for an atretic right kidney. Her ARM index included the cloaca, a normal spine, and sacrum with a lateral sacral ratio of 0.7, predicting good potential for bowel continence. Cystoscopy through the vesicostomy showed a small bladder with normal ureteral orifices, and a closed bladder neck, with no identifiable urethra. A cloacagram showed an atretic common channel, a single small vagina, and a rectum below the pubococcygeal line. The patient underwent a posterior sagittal anorectovaginourethroplasty, vaginal patch using rectum, rectoplasty, and perineal body reconstruction. The urethra was not amenable to reconstruction, so the vesicostomy was preserved and a future Mitrofanoff was planned.

**Conclusion** Urethral atresia is a rare and challenging finding in cloaca patients, and a vesicostomy is needed to drain urine in the newborn period. Preoperative examination under anesthesia, cystoscopy, vaginoscopy, and cloacagram are crucial to identify the precise anatomy and to plan accordingly.

## Introduction

Cloacal malformations comprise a heterogeneous group of anomalies that are considered the most complex anorectal malformations (ARMs) in females. Cloacal malformations are classified according to the lengths of the common channel and the urethra. This classification constitutes the basis whereby the surgeon chooses the surgical approach, either to do a urogenital (UG) separation or a total UG mobilization (TUM). 1 Despite this broad classification, each cloaca case has unique variations that can require modification of the surgical technique to achieve an optimal reconstruction. A thorough preoperative evaluation to identify the specific anatomy of each patient prior to reconstruction, with collaboration between colorectal surgeons, urologists, and gynecologists is vital to plan an individualized approach for each patient. The case presented is a rare anatomical variation in a patient with a cloacal malformation, urethral atresia, a small vagina, and a megarectum, and the reconstructive approach for this patient is described.

## Case Report

**Fig. 1:**
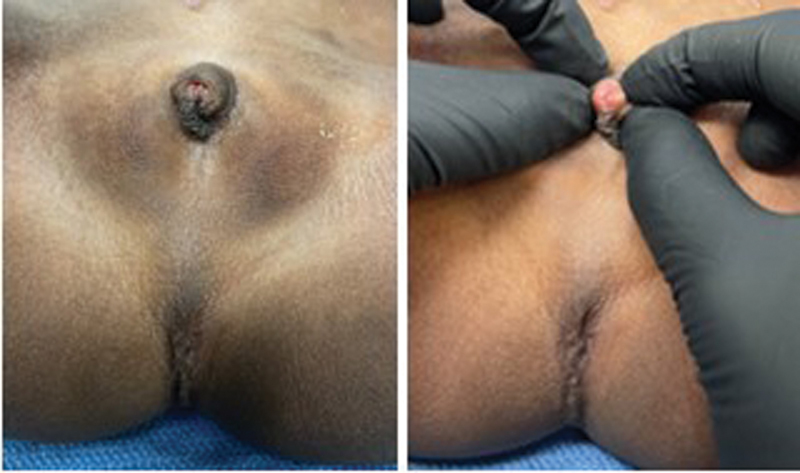
Physical exam reveals rudimentary genital folds, which, when retracted, reveal a small, single perineal opening. There is no anal opening, but there is a gluteal fold with a dimple at the site of anal sphincter.

**Fig. 2:**
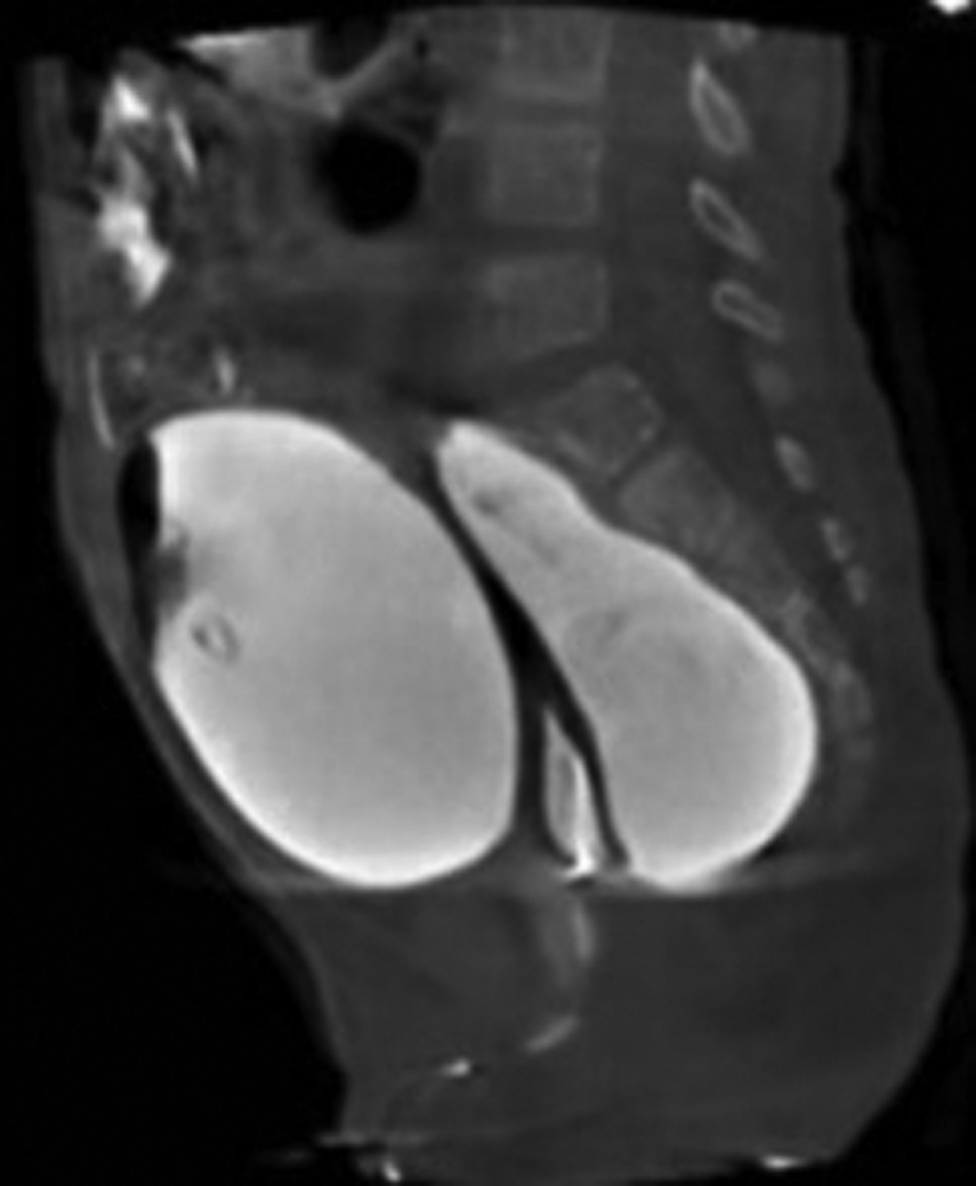
The cloacagram shows an atretic common channel, an atretic urethra, a single small vagina, and a rectum below the pubococcygeal line.

A 6-year-old female with cloaca who underwent colostomy, vaginostomy, and vesicostomy as a newborn was referred to our team for reconstruction. A thorough workup was completed and was significant for the following: The physical examination showed rudimentary genital folds with a single perineal opening, no anal opening, and a gluteal fold with a dimple at the site of the anal sphincter ( Fig. 1 ). The VACTERL workup was negative except for an atretic right kidney and left grade IV vesicoureteral reflux. Her ARM index included the cloaca, a normal spine, and a normal sacrum with a lateral sacral ratio of 0.7, predicting good potential for bowel control. Cystoscopy through the vesicostomy showed a small bladder with a closed bladder neck, normal ureteral orifices, and no identifiable urethra. A cloacagram showed an atretic common channel, a single small and high vagina, and a rectum below the pubococcygeal (PC) line ( Fig. 2 ).

**Fig. 3:**
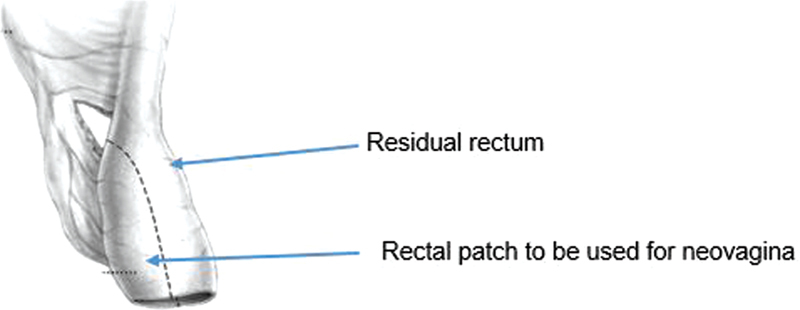
For a rectal patch, the megarectum is divided longitudinally with care to preserve its blood supply.

**Fig. 4:**
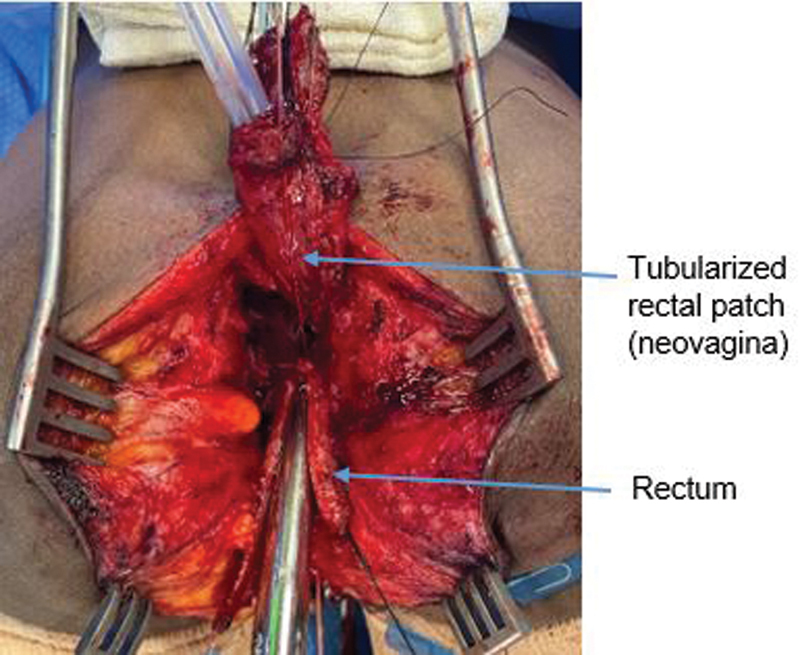
The neovagina is reconstructed using the rectal patch, shown here being tubularized over a Hegar dilator. The patch was sutured to the wall of the vagina circumferentially. A 24-French Foley catheter, used for calibration of the rectoplasty, is seen in the rectum.

The patient underwent a posterior sagittal anorectovaginourethroplasty in prone position. The vagina was small and not amenable to vaginal pull-through. The patient had a megarectum, which allowed for a vaginoplasty using a rectal patch. For the rectal patch creation, we cut a rectangle from the anterior rectal wall based on the two perforating mesenteric vessels on either side ( Fig. 3 ). This created a well-perfused rectangle which could then be sewn to the open vagina, while tubularizing it. We made sure the distal extent reached to the area of the clitoris and was healthy and viable ( Fig. 4 ).

The rectoplasty was done by tubularizing the remnant rectum over a 24-French Foley. An ischiorectal fat pad was used as a cushion between the neovagina and the neorectum. The perineal body reconstruction and introitoplasty were performed in supine position. The urethra was not amenable to reconstruction, therefore the vesicostomy was preserved, with a plan for a future Mitrofanoff. Piperacillin/tazobactam was given as a broad-spectrum prophylactic antibiotic and was continued until the intraoperative urine culture returned as negative. The recovery in the postoperative period was unremarkable. As the procedure had no intra-abdominal component, the patient's diet was resumed immediately following surgery.

The patient is planned for a future colostomy closure with Malone appendicostomy to gain social fecal continence and creation of a continent catheterizable channel for urine (Mitrofanoff), to attain urinary continence.

## Discussion

Cloacal malformations are classified according to the common channel and urethral lengths. This is a broad classification and does not take into account some rare variations such as the case described here. Generally, patients with a shorter common channel length (less than 3 cm) and a longer urethral length (more than 1.5 cm) are amenable to a TUM. Those with a short urethra (less than 1.5 cm) or with common channel length more than 3 cm must undergo a UG separation. 1 In this case, the patient had no urethra or common channel, creating a considerable challenge for reconstruction.

Urethral atresia is rarely mentioned in the literature as most of these patients die in utero or are stillborn. The urethral obstruction in fetuses without drainage of urine leads to oligohydramnios, and pulmonary and renal failure, making this malformation rarely compatible with life. Most of the surviving neonates born with this anomaly have some channel to drain urine antenatally, either through a patent urachus, a vesicocutaneous fistula, a vesical perforation, development of urinary ascites by backflow of urine through the fallopian tubes, or one that is created through an antenatal surgical intervention, like a vesicoamniotic shunt. 2 In the presented case, the presence of a communication between the bladder neck, vagina, and rectum provided a drainage system for urine into the bowel.

Urethral atresia is reported to be associated with prune belly syndrome. One theory suggests that the distended bladder disrupts the development of the abdominal wall musculature. 3 However, an abdominal wall defect was not observed in this patient. In the newborn period the urinary system rarely needs drainage, and ureteral obstruction is almost always due to compression at the trigone by a hydrocolpos. Drainage of the hydrocolpos, usually achieved by catheterization of the common channel almost always succeeds in reducing the hydronephrosis. However, in the case of urethral atresia, the urinary system must be diverted, as was done here using a vesicostomy.

Determining the ideal procedure for the reconstruction in patients with a cloacal malformation is always challenging, but this is particularly true for those with a rare association with urethral atresia, such as in this case. This scenario is discussed in the literature in several instances. González et al reported six male patients with urethral atresia, of whom two patients underwent successful dilation of the urethra followed by rupture of the urethral membrane, and urethral continuity was able to be restored. Continent catheterizable conduits to the bladder were required in four patients, of whom three had undergone unsuccessful attempts at reestablishing urethral continuity by open urethroplasty. 2 Reinberg et al reported a case of a female patient with urethral atresia, vaginal atresia, and imperforate anus. She underwent a neobladder reconstruction, a urethral reconstruction using appendix, a vaginoplasty, and an anoplasty. The patient was continent of urine at age 8 and had normal renal function. 4

Before constructing a continent catheterizable channel, a Mitrofanoff, the family should prove their commitment to frequent catheterization of the infant. Patients without this level of social support should have the Mitrofanoff procedure delayed until they are capable of self-catheterization. In this patient's case, the decision was made to preserve the vesicostomy to drain the urine until the family was prepared to commit to the necessary frequency of catheterizations.

Although this patient has a good ARM index, which predicts a good prognosis for bowel control, the possibility of doing a Malone appendicostomy was discussed. This patient is school age, and the Malone appendicostomy can help this patient to gain social continence and practice gaining control of her anal sphincters using the Malone. This is ideally done at the time of colostomy closure, coordinated with urology team if a Mitrofanoff is needed, to minimize the number of surgical interventions for the patient and to decide how the appendix is divided between the Malone and the Mitrofanoff.

Surgeons operating on complex ARMs, especially cloacal malformations, should be familiar with the different techniques to reconstruct the vagina, to be prepared for when the vagina does not have enough length to reach the perineum. 5 In our center, we do the vaginal reconstruction at the time of the initial surgery, to avoid multiple surgeries on the perineum, which will weaken the area. This would have been more difficult in this patient given the scar tissue and adhesions we had to go through. Ideally, the native vagina is used and bowel replacement is avoided. There is no strict definition on what is a high or low vagina, but for this patient, the vagina ended at the level of the PC line on cloacagram, which we consider as a high vagina. In this patient, it was also small, because it is not dilated from a previous hydrocolpos. For these reasons, it was unlikely it would have been able to be mobilized to reach the perineum without tension.

In such rare cases, in order to bridge the gap of a vagina that does not reach the perineum, the options include vaginal replacement using small bowel, large bowel, or rectum. Using a rectal patch is a good option to reconstruct the vagina when the rectum is large enough to be used for both the vaginoplasty and the rectoplasty, as in this patient's case, due to her megarectum. 6 A large rectum does not have normal motility and thus, reducing its size might be helpful to approach a more normal rectal capacity. Having a more normal-sized rectum will help to optimize the patient's motility and improve their chances for bowel continence. The rectum in this case was reconstructed by reducing its size and tubularizing it over a 24-French Foley catheter to mimic a normal rectum.

## Conclusion

This case illustrates the importance of careful identification of the precise anatomy in each patient with cloaca and highlights the importance of having different reconstructive techniques at the surgeon's disposal to optimize the pelvic reconstruction, tailored to the particular patient's needs.

## Notes

New Insights and the Importance for the Pediatric Surgeon In this case, a patient with rare anomaly—cloaca with urethral atresia—is presented, highlighting the importance of preoperative investigations to understand the anatomy to plan the best surgical management. It is rare that the urinary system in a cloaca must be diverted in the newborn period, but urethral atresia is one such situation.
